# Distribution, associations and role in the biological carbon pump of *Pyrosoma atlanticum* (Tunicata, Thaliacea) off Cabo Verde, NE Atlantic

**DOI:** 10.1038/s41598-021-88208-5

**Published:** 2021-04-29

**Authors:** Vanessa I. Stenvers, Helena Hauss, Karen J. Osborn, Philipp Neitzel, Véronique Merten, Stella Scheer, Bruce H. Robison, Rui Freitas, Henk Jan T. Hoving

**Affiliations:** 1 GEOMAR, Helmholtz Centre for Ocean Research Kiel, Düsternbrooker Weg 20, 24105 Kiel, Germany; 2 Department of Invertebrate Zoology, National Museum of Natural History, Smithsonian Institution, Washington, DC 20013 USA; 3 Faculty of Science and Engineering, University of Groningen, Nijenborgh 4, 9747 AG Groningen, The Netherlands; 4 Monterey Bay Aquarium Research Institute, 7700 Sandholdt Road, Moss Landing, CA 95039-9644 USA; 5 Institute of Engineering and Marine Sciences, Atlantic Technical University, CP 163, Mindelo, Cabo Verde

**Keywords:** Ecology, Zoology, Ecology, Ocean sciences, Marine biology, Biogeochemistry, Carbon cycle

## Abstract

Gelatinous zooplankton are increasingly acknowledged to contribute significantly to the carbon cycle worldwide, yet many taxa within this diverse group remain poorly studied. Here, we investigate the pelagic tunicate *Pyrosoma atlanticum* in the waters surrounding the Cabo Verde Archipelago. By using a combination of pelagic and benthic in situ observations, sampling, and molecular genetic analyses (barcoding, eDNA), we reveal that: *P. atlanticum* abundance is most likely driven by local island-induced productivity, that it substantially contributes to the organic carbon export flux and is part of a diverse range of biological interactions. Downward migrating pyrosomes actively transported an estimated 13% of their fecal pellets below the mixed layer, equaling a carbon flux of 1.96–64.55 mg C m^−2^ day^−1^. We show that analysis of eDNA can detect pyrosome material beyond their migration range, suggesting that pyrosomes have ecological impacts below the upper water column. Moribund *P. atlanticum* colonies contributed an average of 15.09 ± 17.89 (s.d.) mg C m^−2^ to the carbon flux reaching the island benthic slopes. Our pelagic in situ observations further show that *P. atlanticum* formed an abundant substrate in the water column (reaching up to 0.28 m^2^ substrate area per m^2^), with animals using pyrosomes for settlement, as a shelter and/or a food source. In total, twelve taxa from four phyla were observed to interact with pyrosomes in the midwater and on the benthos.

## Introduction

Although gelatinous zooplankton are among the most abundant inhabitants of the open ocean^1,2^, their roles in marine ecosystems have traditionally been underestimated^3^. Gelatinous zooplankton refers to a polyphyletic group of marine organisms, characterized by high water content of their tissues (~ 95%) and a planktonic existence (carried by the currents). This group includes taxa such as ctenophores, medusae, siphonophores and pelagic tunicates (i.e. salps, pyrosomes, doliolids and larvaceans)^1,3,4^. Since many of these animals possess delicate bodies that are easily damaged by net collections, it was not until the advent of underwater technologies such as blue-water SCUBA and submersibles that their ability to seasonally dominate pelagic midwater communities was noted^1,3,4^. With this discovery, a more complex picture of their roles in marine ecosystems soon emerged. Gelatinous zooplankton are now increasingly recognized as important players in the global carbon cycle, accumulating and transporting organic carbon to the seabed^5–7^. Nevertheless, at present day, only a small fraction of our ocean has been explored and gelatinous zooplankton remain poorly studied, particularly in deep pelagic ecosystems^2,8^.

Pyrosomes are abundant gelatinous zooplankton in open ocean environments and continental shelf slopes, but relatively little is known about their general biology^9–12^. Most studies on the role of pyrosomes in ocean ecosystems focus on *Pyrosoma atlanticum* Peron, 1804, a species common throughout the Pacific, Atlantic and Indian Oceans^13^. *Pyrosoma atlanticum* has been shown to markedly redirect primary production with clearance rates measured up to 35 L h^−1^ for a single colony^12^. Additionally, dense shoals were estimated to clear up to 53% of the phytoplankton stock^14^. Since *P. atlanticum* possesses one of the highest carbon contents measured among gelatinous zooplankton (35% of dry weight)^15^, sinking moribund or dead colonies can transport substantial amounts of organic matter to the deep^15–17^. Mass mortality of pyrosome blooms, for instance, can result in so called ‘jelly-falls’, providing abundant food for a variety of benthic fauna^15,17^. Together with living pyrosomes, decrepit colonies were shown to be consumed by echinoderms, actinarians, crustaceans^15,17^, fishes, turtles^18^, marine birds^19,20^ and even marine mammals^21,22^. Few of these studies, however, have addressed the ecology of *P. atlanticum* in the water column, and rarely via in situ observations^15–17,23^.

In addition to being a food source, living pyrosome colonies further add to the downward flux of carbon by the fast production of fecal pellets, which may have as much as 22% carbon per unit dry weight^14^. The rate of this downward carbon flux may be further enhanced through *P. atlanticum*’s diel vertical migrations, by which they can actively transport fecal material below the mixed layer^10^. The extent of this transport, however, has only been estimated theoretically^10,14^ and it remains unknown whether the fecal material reaches greater depths in practice. To our knowledge, pyrosome fecal pellets have not been reported from sediment traps, even though the sedimentation of salp fecal material has been well documented (e.g.^24,25^ and references therein). A novel molecular method that may provide an effective tool to confirm this transport is the detection and barcoding of environmental DNA (eDNA)^26^. eDNA is defined as the genetic material that organisms shed in the form of dead tissue cells, feces or mucous, which can be extracted from their environment as it usually remains in the water for a period of time without direct presence of the animal. Although eDNA analyses are becoming increasingly popular to document biodiversity of marine communities^26,27^, the technique has never been implemented to detect pyrosome DNA (from colonies or fecal pellets) at depth.

During two research cruises to the Cabo Verde region of the eastern tropical North Atlantic (ETNA), we encountered large aggregations of *P. atlanticum,* which allowed us to investigate its ecological role in this system. Even though the open ocean surrounding the Cabo Verde archipelago is oligotrophic, geographic features such as seamounts and islands induce local upwelling and therefore enhance biological productivity^28,29^. In addition, northeasterly trade winds force eddy formation in the wake of the islands and enhance upwelling depending on season^30,31^. The region features a weak mesopelagic oxygen minimum zone (OMZ), centered around 450 m depth with dissolved oxygen concentrations reaching ~ 40 µmol kg^−1^^32,33^. By combining multinet sampling with genetic molecular tools and in situ video observations from a manned submersible and two towed camera platforms, our aim was to investigate: (i) the abundance and vertical distribution of *P. atlanticum* in the water column in relation to environmental drivers, (ii) its role in the transport of organic carbon, and (iii) associations with other pelagic and benthic organisms.

## Methods

### Sampling stations

**Figure 1:**
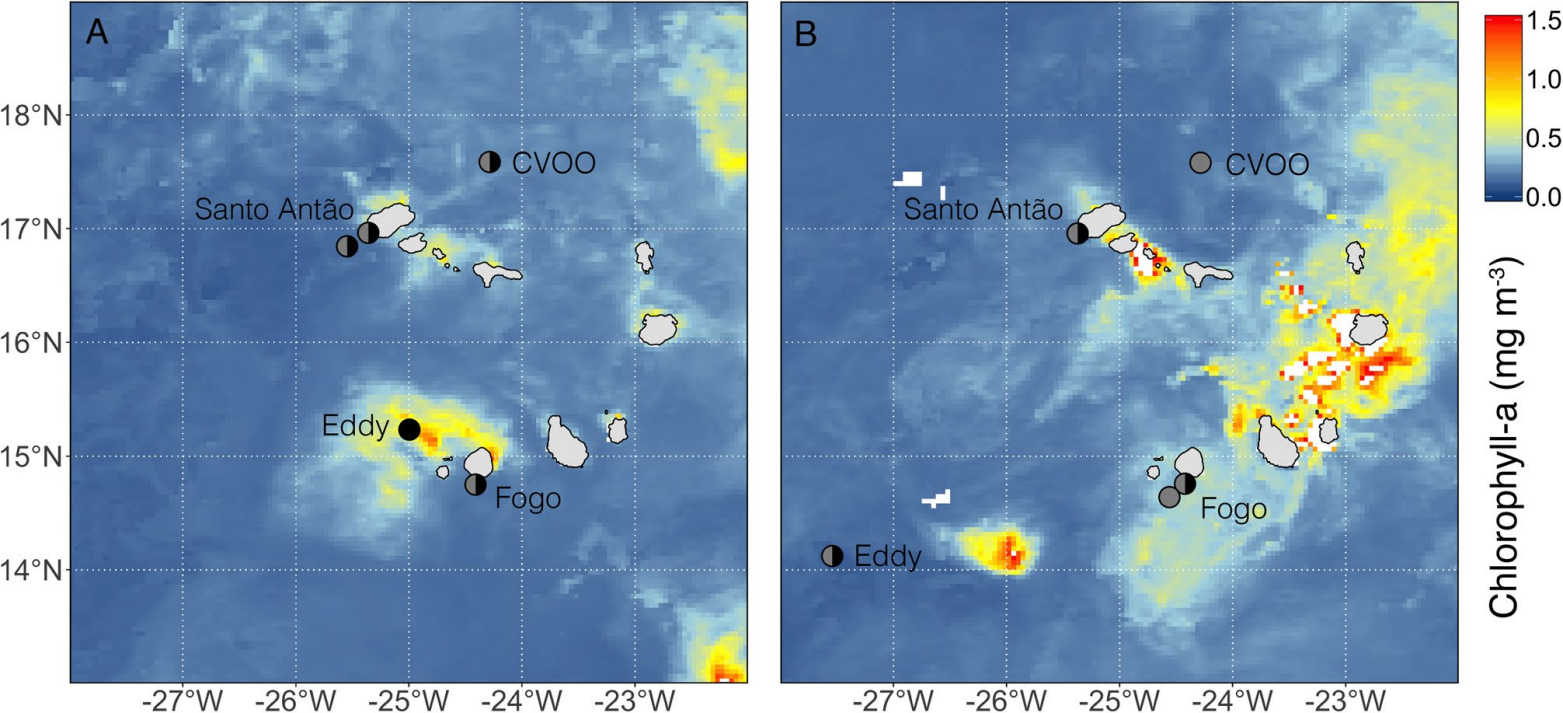
Sampling stations during the (**A**) POS520 cruise in 2018 and the (**B**) POS532 cruise in 2019, showing respective mean monthly chl-a concentrations (mg m^−3^) from the MODIS-aqua database^35^. Since cloud cover obstructed chl-a measurements in February for both years, monthly measurements are shown for March in 2018 and January in 2019. As a result, the MODIS-aqua data does not align with our cyclonic eddy sampling points. White fields indicate missing data due to cloud cover. Circles indicate day (grey) and night time (black) sampling.

*Pyrosoma atlanticum* were sampled during two R/V *Poseidon* research cruises from February 14–March 1, 2018 (POS520) and February 4–24, 2019 (POS532). Sampling stations were located off the Cabo Verde Archipelago and included the Cabo Verde Ocean Observatory (CVOO)^34^ in the open ocean windward of the islands, two cyclonic eddy cores, and coastal and oceanic deployment sites in the lees of the islands of Santo Antão and Fogo (Fig. 1). Most stations were sampled during both day and night, with the exceptions of the eddy station in 2018, CVOO in 2019, and the oceanic station near Fogo in 2019, which were only sampled during the day. Eddies were identified and tracked with the help of satellite altimetry based on their negative sea level anomaly, reduced sea surface temperature and enhanced chlorophyll-*a* (chl-*a*) concentrations^31^. Eddy cores were targeted using the ship’s thermosalinograph (TSG) and acoustic Doppler current profiler (ADCP). CTD profiles (i.e. temperature, salinity, oxygen and chl-*a* concentrations) were collected at each sampling station, while additional chl-*a* surface concentrations were obtained from the Moderate Resolution Imaging Spectroradiometer (MODIS) aqua database^35^.

To confirm the depth range of pyrosome material, and to learn if the presence of pyrosomes can be confirmed through molecular methods, eDNA was sampled at varying depths in the cyclonic eddy (at 400, 600, 1000, 1900, 2200 and 2500 m) and CVOO station (at 1000, 1300, 1900, 2500 m) in 2019. The latter acted as a reference station where we did not observe pyrosomes. Water samples for the eDNA analyses were collected with 10 L Niskin bottles attached to a CTD rosette, while on board each depth was sampled in biological triplicates consisting of 2 L seawater. Samples were immediately filtered by letting the water drip through filters (0.22 µm pore size sterivex™ filters MERCK), which were then stored at -80 °C. Back in the laboratory ashore, samples were barcoded for pyrosome eDNA as described in the Supplementary Material.

Cabo Verde has not ratified the Nagoya protocol. To fulfill the national ABS regulations of Cabo Verde, we obtained the required permit for the publication of results based on samples collected in Cabo Verde waters from the Direcção Nacional do Ambiente (National Directorate for the Environment of Cabo Verde).

### Vertical depth distribution, abundance and substrate area

The vertical distribution and abundance of *P. atlanticum* were investigated using two methods. First, oblique multinet hauls were used to collect *P. atlanticum* colonies at different depth strata (Supplementary Table S1) using a Hydrobios© Maxi multinet (0.5 m^2^ in aperture, 2 mm mesh size, nine nets and electronic flow meters) towed at approximately 2 kn. Pyrosome length, width and wet weight were quantified as described in the Supplementary Methods. The weighted mean depth (WMD) for each haul was calculated using the WW of pyrosomes per multinet depth bin (g) and median depth (*d* in meters) of each bin per sampling station (*i*):

1 $$\begin{array}{*{20}c} {WMDi = \frac{{\sum \left( {WWi \cdot di} \right)}}{\sum WWi}} \\ \end{array}$$

Second, the towed camera platform Pelagic In situ Observation System (PELAGIOS)^36^ was deployed to record quantitative video footage of pyrosomes at various depths (Supplementary Table S2). PELAGIOS was towed in a stair-step trajectory at the ship’s average speed of approximately 0.51 m s^−1^, resulting in horizontal and vertical video transects. The sampling volume (2.54 m^3^ s^−1^) was estimated by mounting the PELAGIOS camera to the multinet (i.e. the MuViNet) at the Fogo and Eddy stations in 2018 and comparing video counts and net catches using a linear model following calculations by Hoving et al*.*^36^ (Supplementary Figure S1). Video recordings were annotated using the Video Annotation and Reference System (VARS) developed by the Monterey Bay Aquarium Research Institute^37^.

The depth-integrated substrate area provided by *P. atlanticum* aggregations was calculated per station as described in the Supplementary Methods.

### Fecal pellet production and respiratory carbon flux

The downward active carbon flux from *P. atlanticum* fecal pellet production, linking primary production to depth, was based on nighttime multinet biomass. Since we did not catch any pyrosomes above the mixed layer during the day, we assume that all *P. atlanticum* migrated downward. The fecal pellet production (FP; in mg C colony^−1^ day^−1^) was calculated following the formula by Henschke et al*.*^10^ based on observations from Drits et al.^14^:

2 $$\begin{array}{*{20}c} {FP = 0.25 \cdot CC} \\ \end{array}$$

where CC is the carbon content of *P. atlanticum* WW biomass (i.e. 3.92% of WW)^10,15^.

To calculate the number of fecal pellets that are actively transported by migrating colonies, we used the gut turnover rate (GTR i.e. the time it takes for *P. atlanticum* to digest food particles, in hours) and the time needed to vertically migrate below the mixed layer (DM in hours). The GTR was obtained from Perissinotto et al*.*^12^, who reported a gut processing time of 1.43 h for *P. atlanticum* at presumably 13.7 to 17.8 °C, which, although not directly reported, was the temperature range for all other experiments in their study, and similar to the temperatures measured here. Since temperature is known to affect metabolic rate, we chose not to use the GTR reported by O’Loughlin et al.^38^, who measured a GTR of 2.6 h for *P. atlanticum* at a much colder 12 °C. Additionally, the faster GTR by Perissinotto et al*.*^12^ results in most conservative estimates for the fecal pellet carbon flux (FPF) as it implies that most gut content will be evacuated before migrating out of the productive upper water layers. The DM was calculated based on the swimming speed of 0.05 m s^−1^ reported by Henschke et al*.*^10^ and the distance from the average night WMD to the mixed layer depth at approx. 100 m (i.e. 54.2 m). The active fecal pellet carbon flux (FPF in mg C colony^−1^ day^−1^) was calculated as follows^10^:

3 $$\begin{array}{*{20}c} {FPF = \frac{FP}{{24}} \cdot \left( {1.43 - DM} \right)} \\ \end{array}$$

To calculate hourly rates from daily rates, the FP was divided by 24 h. To obtain the total amount of fecal pellet carbon produced in the mixed layer at night, the hourly rates were multiplied by 8.5, which was the approximate time spent near the surface.

Besides the fecal pellet flux, pyrosomes actively transport carbon below the mixed layer through the respiratory release of carbon dioxide^10^. This respiratory flux was quantified from daytime multinet biomass when pyrosomes were present below the mixed layer. For this, the respiratory carbon equivalent (RC, in µg C m^−2^ day^−1^) was calculated according to the formula by Al-Mutairi and Landry^39^ using the respiration rate (R, see Eq. (5)), the respiratory quotient (RQ, i.e. 1.16)^40^, the molar weight of carbon (12) and the molar volume of an ideal gas at standard pressure and temperature (22.4):

4 $$\begin{array}{*{20}c} {RC = R \cdot RQ \cdot \left( {\frac{12}{{22.4}}} \right)} \\ \end{array}$$

The R and RQ terms were derived from Henschke et al*.*^10^. Since there is no respiratory quotient for pyrosomes available, the respiratory quotient for salps was used. The oxygen respiration (in ml O_2_ colony^−1^ hour^−1^) for *P. atlanticum* was calculated as follows, using the average WW (g) per station:

5 $$\begin{array}{*{20}c} {R = 0.0046WW^{1.2284} } \\ \end{array}$$

To get to the final respiratory carbon equivalent, the respiration rate was multiplied by the time spent below the mixed layer and the depth-integrated abundance.

### Organic carbon transport and biological associations

The instantaneous organic carbon flux as a result of pyrosome mortality was estimated by quantifying carcasses on the seabed at the Santo Antão and Fogo stations in 2019. High definition video recordings of these pyrosome-falls were made with the manned submersible JAGO^41^ and the towed Ocean Floor Observation System (OFOS) equipped with the same camera and telemetry as PELAGIOS^36^, two lasers for size reference, and a CTD for depth recording. OFOS was towed just above the seabed between 1500 to 200 m depth at the ship’s average speed of 0.51 m s^−1^. JAGO was restricted to < 400 m depth and either drifted with the currents or motored purposely to qualitatively investigate species associated with pyrosomes in the water column and on the seafloor. JAGO was also deployed in 2018 for additional midwater surveys. Observations of crustaceans and other animals associated with pyrosomes were made using all three platforms. To identify some of the crustaceans and the tissue they were attached to, select specimens were sampled with the suction samplers or acrylic collecting cylinders mounted to JAGO. Live animals were photographed on board using a Canon EOS 5DS R camera with a Canon 65 mm f/2.8 1–5 × macro lens. In the laboratory ashore, the samples were barcoded for DNA as described in the Supplementary Methods. The video recordings were annotated using VARS as above for midwater observations^37^. Next, carbon content (CC; C in µg) of the pyrosome carcasses was calculated following the equation by Lavaniegos and Ohman^42^ using the average total length of colonies in 2019 (TL, 80.9 ± 16.4 mm):

6 $$\begin{array}{*{20}c} {CC = 12.54TL^{1.90} } \\ \end{array}$$

Bathymetric data from the Cabo Verde region were obtained from the GEBCO database^43^ and used to plot the relative concentrations of pyrosomes on the seabed.

### Statistical analysis

To investigate what environmental conditions led to increased *P. atlanticum* abundance, both linear and median quantile regression were used respectively for the multinet spatial and vertical distributions. Since the latter dataset deviated from normality, a non-parametric model was implemented. At the stations and depths where no pyrosomes were caught in the multinets, absence was recorded as zero values in both models. In the spatial model, depth-integrated abundance was tested against average sea surface temperature, minimum oxygen concentrations and depth-integrated chl-*a* concentrations at each station. In the vertical distribution model, the integrated abundance per depth stratum was correlated to the latter variables recorded at mid-depth of each depth bin, introducing day and nighttime observations as nested factor. Due to large differences in absolute abundance, the integrated abundance per depth stratum was normalized as a fraction of the total integrated abundance per station. In both analyses, the Akaike’s Information Criterion (AIC) was used to determine the most parsimonious model based on combinations and single terms of the variables. All data were analyzed using R 3.5.2.

## Results

### Oceanographic conditions

All sampling stations were characterized by relatively high surface temperatures, ranging between 17.5 ± 2.9 (s.d.) and 22.1 ± 1.1 °C in the upper 100 m of the water column for both years (Supplementary Table S3). Below the thermocline, these temperatures gradually decreased to reach ~ 6 °C around 400 m depth (Supplementary Figure S2). Lowest dissolved oxygen concentrations were recorded between 300–400 m depth (i.e. the OMZ core), with a minimum concentration of 42.8 µmol kg^−1^. The mean monthly chl-*a* data from the MODIS-aqua database (Fig. 1) showed that the water masses surrounding the Cabo Verde Islands were characterized by relatively high productivity. In both years, the highest integrated (upper 100 m) chl-*a* concentrations were measured in the eddy during the night (reaching 46.0 mg m^−2^ in 2018 and 44 mg m^−2^ in 2019) and lowest at the CVOO during the day (dropping to 21.0 mg m^−2^ in 2018 and 16.4 mg m^−2^ in 2019; Supplementary Table S3).

Although non-significant, the most parsimonious model to explain *P. atlanticum* spatial distribution (using integrated abundance) included sea surface temperature as the main driver of abundance (Linear regression, R^2^_adj._ = 0.170, df = 13, F = 3.856, *p* = 0.071; Supplementary Figure S3A and Table S4). Here, lower temperatures correlated with greater pyrosome abundances. For the vertical distribution, the most parsimonious model only included chl-*a*, with increased chl-*a* concentrations correlating significantly to increased abundance at night (Median quantile regression, chl-*a* vs. nighttime abundance, df = 4, *p* < 0.000; Supplementary Figure S3B and Table S4).

### Vertical distribution, abundance and substrate area

**Figure 2:**
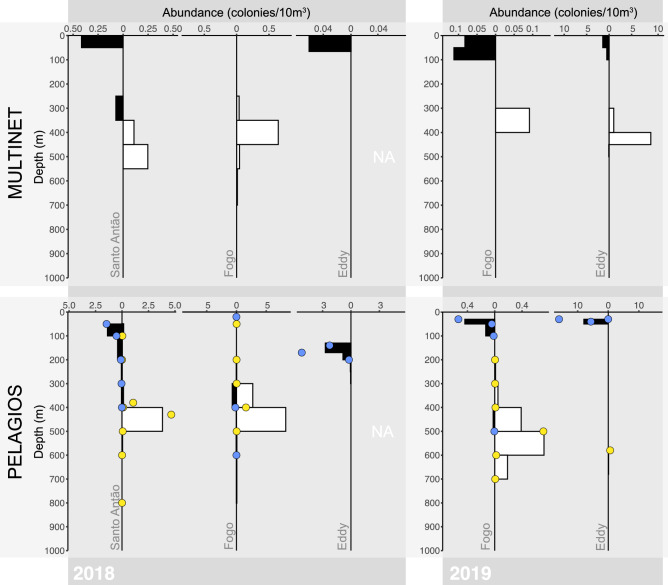
Vertical distribution of *P. atlanticum* (colonies 10 m^−3^) in the eastern tropical North Atlantic, as caught with the multinet (upper plots) and observed with PELAGIOS (lower plots) in 2018 (left) and 2019 (right). Observations were made at Santo Antão, Fogo and two cyclonic eddies. For the PELAGIOS observations, bars indicate abundance counted during vertical descents of PELAGIOS, while dots indicate horizontal transects. Both day (yellow dots, white bars) and nighttime (blue dots, black bars) observations are shown.

*Pyrosoma atlanticum* was recorded at all sampling stations with exception of the CVOO in 2018, and was only observed in the cyclonic eddy and at the coastal Fogo station in 2019. During the day, *P. atlanticum* was present below the mixed layer (mixed layer depth 100 m), while most colonies migrated to the surface at night (Fig. 2). During these diel vertical migrations, pyrosomes migrated on average 313 m, with an average daytime weighed mean depth of 359 m and 46 m at night (Supplementary Table S5). It should be noted that the daytime distribution coincided with the OMZ core between 300 and 400 m depth. The deepest pyrosomes observed were recorded with PELAGIOS at the Santo Antão oceanic night station in 2018, where seven colonies were observed between 1300 and 2500 m depth. These colonies did not have the straight cylindrical shape typical for most pyrosomes, but instead were bent or almost spherical. They did have a similar color to living pyrosomes as they were semi-transparent pink to purple, rather than the opaque grey frequently observed in dead colonies.

The multinet and PELAGIOS platforms recorded slightly different depth-integrated abundance and densities, with PELAGIOS generally observing higher numbers of colonies (Supplementary Table S6, Fig. 2). In 2018, PELAGIOS observed highest depth-integrated abundances at the coastal Fogo station (110.51 and 8.37 colonies m^−2^ during the day and night, respectively), with maximum concentrations of 8.27 colonies 10 m^−3^ between 400 and 500 m (day). The multinet data, on the other hand, showed highest abundances at the Santo Antão oceanic station in 2018 (17.85 colonies m^−2^ at night, no daytime numbers available), but maximum concentrations of 0.64 colonies⋅10 m^−3^ between 350 and 450 m (day) at the Fogo station. Surprisingly, the multinet did not catch any pyrosomes during the night at Fogo, while PELAGIOS’ ‘night’ sampling had already commenced at 16:30 h (UTC) with maximum densities reaching only 0.72 colonies⋅10 m^−3^ between 300 and 400 m. In 2019, PELAGIOS observed maximum abundances in the cyclonic eddy (1.07 and 15.93 colonies m^−2^ during the day and night, respectively), with maximum densities of 16.07 colonies 10 m^−3^ at 30 m (night). At the same station, the multinet showed 56.75 and 10.12 colonies m^−2^ during the day and night, respectively, with maximum concentrations reaching 9.28 colonies 10 m^−3^ between 400 and 450 m (day). The horizontal distribution of *P. atlanticum* was heterogeneous and neither multinet nor PELAGIOS ever recorded a similar abundance at a station twice.

The depth-integrated substrate area provided by the pyrosome aggregations ranged between 17.0–368.0 cm^2^ per m^2^ water surface in 2018 and 28.3–2820.4 cm^2^ m^2^ in 2019 (Supplementary Table S6).

### Fecal pellet production, respiratory carbon flux and eDNA

The production of fecal pellets at night in the mixed layer accounted for an estimated 14.75–295.51 mg C m^−2^ day^−1^ in 2018 and 19.12–485.57 mg C m^−2^ day^−1^ in 2019 (Supplementary Table S5). It was estimated that, during the downward migration at dawn, pyrosomes transport 13.3% of these fecal pellets below the mixed layer, corresponding to 1.96 to 64.55 mg C m^−2^ day^−1^ (Supplementary Table S5).

The amount of respiratory carbon released below the mixed layer accounted for only 0.0003 to 0.0053 mg C m^−2^ day^−1^ in 2018 and 0.0004 to 0.0393 mg C m^−2^ day^−1^ in 2019 (Supplementary Table S5). Since the oceanic station in the lee of Santo Antão and the Eddy in 2018 only included nighttime sampling, the respiratory carbon flux for these stations was based on nighttime biomass, as we assume that these pyrosomes migrated below the mixed layer given our observations from other stations (Fig. 2). The respiratory carbon flux accounted for approx. 0.03 ± 0.02% of the fecal pellet flux.

Our eDNA analysis showed that the presence or traces of pyrosomes can be detected in water samples (Supplementary Figure S4), although we only detected four sequences in total. In the cyclonic eddy in 2019, the station of maximum pyrosome abundance, water samples collected at 400 m (n = 2), 600 m (n = 1) and 1000 m (n = 1) had a 100% Blastn match with 100% coverage to sequences from *P. atlanticum*, *Pyrostremma spinosum* (Herdman, 1888), *Pyrosomella verticillata* (Neumann, 1909) and *Pyrosoma godeauxi* van Soest, 1981 (Supplementary Table S7). In comparison, PELAGIOS only recorded pyrosome colonies down to 700 m and the multinet caught colonies down to 500 m in the cyclonic eddy (Fig. 2). No pyrosome eDNA was detected at the CVOO, which is in correspondence with our observational data.

### Organic carbon on the seabed

**Figure 3:**
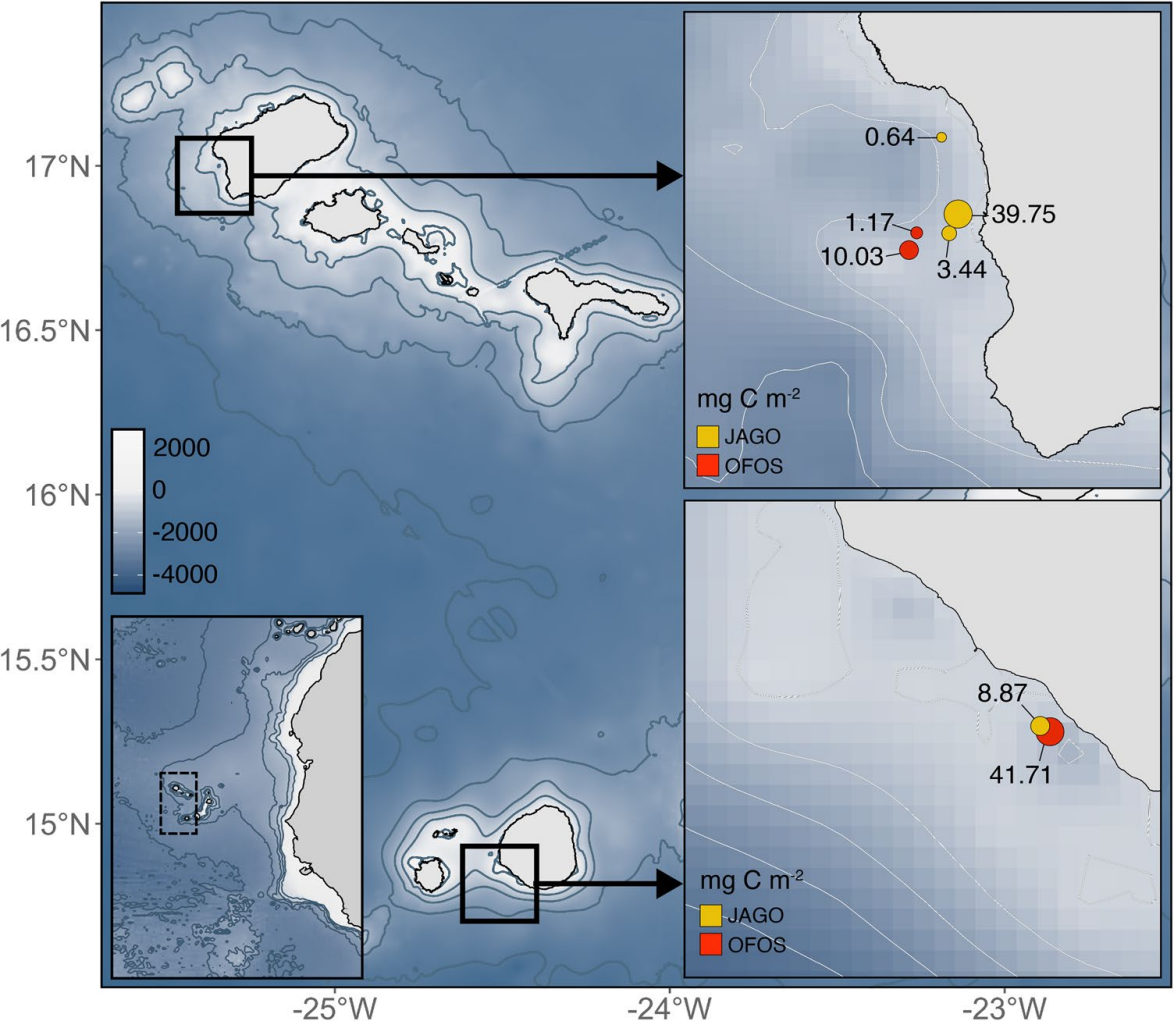
Carbon depositions of *P. atlanticum* in mg C m^−2^ near Cabo Verde Archipelago (left lower panel, dashed line) on island slopes of Santo Antão (right upper panel) and Fogo (right lower panel). Observations were made with the manned submersible JAGO (yellow) and towed camera platform OFOS (red). Bathymetric data was obtained from the GEBCO database^43^.

All *P. atlanticum* carcasses were found on the shallower end of the island slopes (Fig. 3), with a total of 404 pyrosomes observed at Santo Antão and 140 at Fogo (Supplementary Table S8). The deposited pyrosomes observed between 500 and 213 m had a similar color to living colonies (i.e. pink to purple), with many more floating > 1–2 m above the seabed (i.e. 133 seen at Santo Antão and 136 at Fogo). Relative carbon depositions were similar at both locations, accounting for up to 41.71 and 39.75 mg C m^−2^ at the Fogo and Santo Antão stations, respectively (Fig. 3, Supplementary Table S8).

### Biological associations

**Figure 4:**
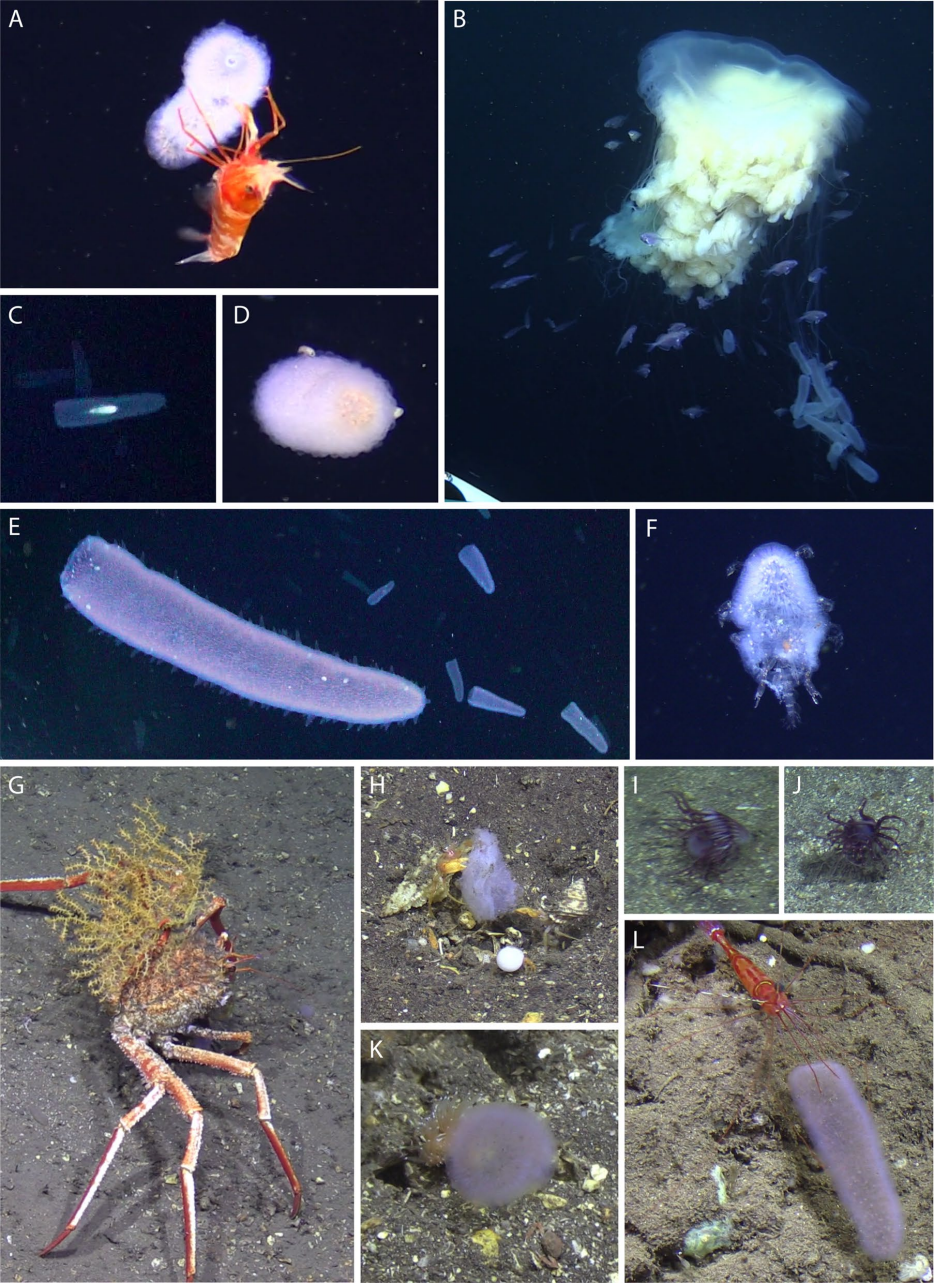
Pelagic (**A**–**F**) and benthic (**G**–**L**) animals associated with *P. atlanticum* in the eastern tropical North Atlantic, observed in February 2018 and 2019. (**A**) Unidentified Oplophoridae and (**B**) *Drymonema gorgo* feeding on pyrosomes. (**C**) An unidentified fish swimming inside pyrosome. (**D**,**E**) Unidentified hyperiid amphipods and (**F**) *Phronima* sp. using *P. atlanticum* as substrate, potentially feeding and reproducing on it. (**G**) An unidentified decorator crab, (**H**) three unidentified Anomura, (**I**,**J**) two unidentified Actiniaria, (**K**) *Dondice* sp. and (**L**) *Pandalopsis* sp. feeding on pyrosomes. All images were recorded with JAGO with exception of (**C**) and (**E**), which were recorded with PELAGIOS.

**Figure 5:**
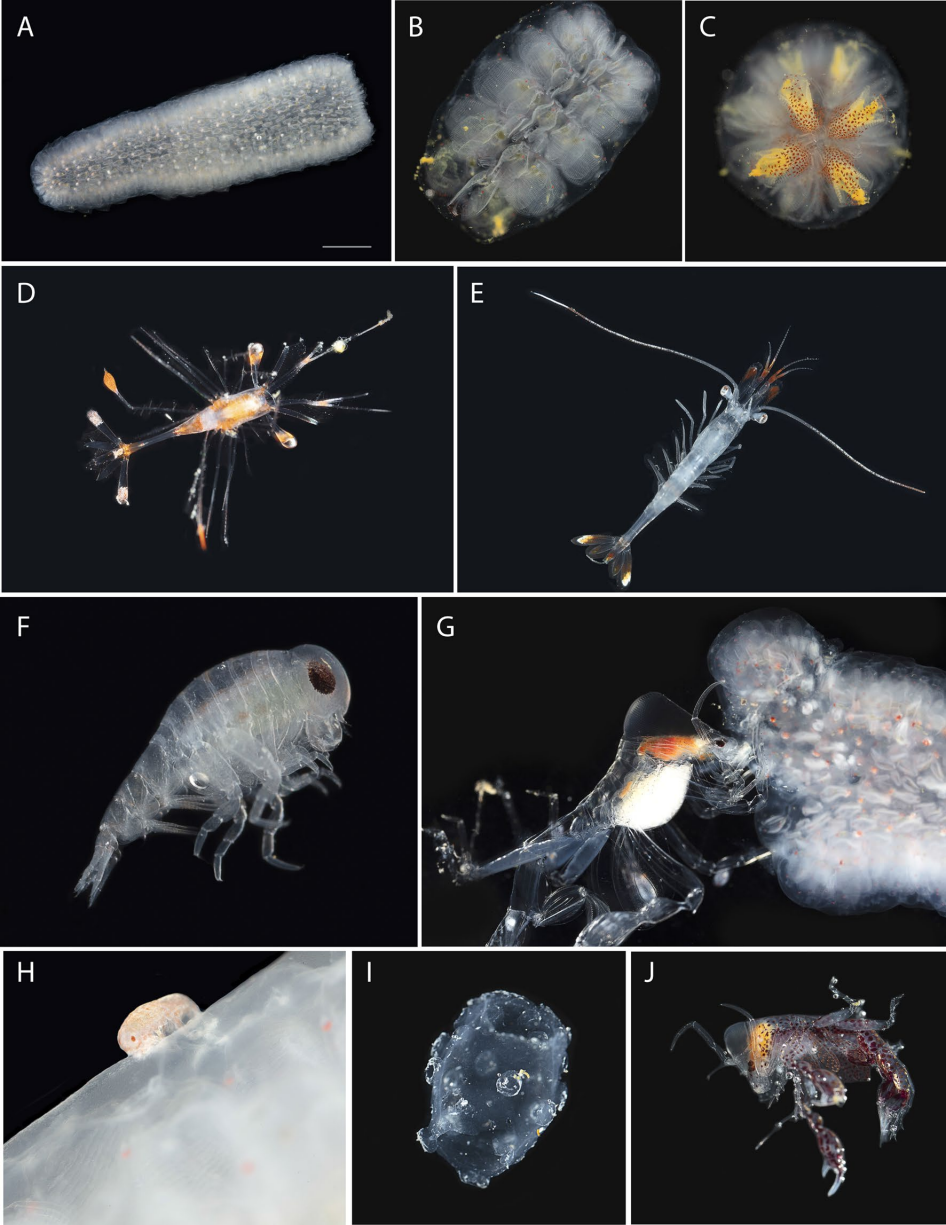
Macro photographs of live animals on board R/V *Poseidon*. (**A**–**C**) *P. atlanticum*, scale bar 1 cm. Individual zooids visible in B and C, colonies respectively ~ 1 cm in length and < 3 mm in diameter. (**D**,**E**) Early (< 1 cm) and late (> 2 cm) juvenile *Funchalia villosa* collected from *P. atlanticum*. (**F**) Unknown juvenile oxycephalid collected off *P. atlanticum,* body length < 2 mm. (**G**) *Phronima* specimen feeding on *P. atlanticum* colony overnight and undeterred by the camera strobes. (**H**) Unknown juvenile oxycephalid still attached to *P. atlanticum*, body length < 1 mm. (**I**–**J**) *Phronima*-modified barrel made from a *P. atlanticum* colony that was inhabited by the individual in J, barrel was just under 2 cm in length.

Different organisms were observed to interact with *P. atlanticum* in the water column and on the seabed, most likely involving feeding (Table 1). In the pelagic realm, eleven unidentified shrimps of the Oplophoridae Dana, 1852 (220–360 m, Fig. 4A) and the medusa *Drymonema gorgo* Müller, 1883 (50 m, Fig. 4B) were seen feeding on *P. atlanticum*. Juvenile stages of the penaeid shrimp were observed within and on pyrosomes (Fig. 5D–E). These shrimps have distinctive red and white patterns on their antennae, telson and uropods and were often observed inside a colony with antennae projecting. DNA barcoding identified them as *Funchalia villosa* (Bouvier, 1905) (COI 92–100% coverage; voucher USNM1524835). Furthermore, PELAGIOS in situ observations revealed four occasions where a small fish was situated inside *P. atlanticum* (20–30 m, Fig. 4C).

**Table 1:** Biological associations of *P. atlanticum* in the eastern tropical North Atlantic.

Species	Association	Type	Date	Start dive (UTC)	Station	Depth (m)
**Chordata**						
Unidentified Actinopterygii	Swimming inside *P. atlanticum,* observed 4 times	Pelagic: trophic, shelter	22/02/18,22/02/19	21:21, 21:04	Fogo, Eddy	20, 30
**Crustacea**						
Unidentified Oplophoridae	Sitting on and eating *P. atlanticum*, observed 11 (2018) and 5 (2019) times	Pelagic: substrate, trophic	22/02/18,17/02/19, 18/02/19	09:28, 15:56, 07:15	Fogo	220–300, 290–330, 360
*Funchalia villosa*	Sitting inside and on *P. atlanticum*	Pelagic: substrate	18/02/19	07:15	Fogo	320
*Pandalopsis* sp.	Eating *P. atlanticum*, observed 5 times	Benthic: trophic	17/02/19	15:56	Fogo	230
*Phronima* sp.	Holding cut *P. atlanticum*	Pelagic: trophic, reproduction	08/02/19	21:30	Santo Antão	70
*Primno* sp.	Attached to *P. atlanticum* on seafloor, observed 2 times	Benthic: trophic	10/02/19	09:24	Santo Antão	312
*Hyperiid amphipods*	Holding, sitting/swimming in and on *P. atlanticum.* (i.e. including *Hyperia* and Oxycephalidae)	Pelagic: Substrate, trophic	All	–	All	All
Unidentified Decorator crab	Eating *P. atlanticu*	Benthic: trophic	10/02/19	09:24	Santo Antão	382
Unidentified Anomura	Five *Anomura eating* *P. atlanticum*	Benthic: trophic	12/02/19	09:16	Santo Antão	361
**Mollusca**						
*Dondice* sp.	Sitting on *P. atlanticum*	Benthic: trophic	10/02/19	09:24	Santo Antão	315
Unidentified Gastropod	Eating *P. atlanticum*	Benthic: trophic	17/02/19	15:56	Fogo	307
**Cnidaria**						
*Drymonema gorgo*	Twelve *P. atlanticum* caught in tentacles	Pelagic: trophic	26/02/18	09:14	Fogo	50
Unidentified Actiniaria	Eating *P. atlanticum*, observed 2 times	Benthic: trophic	10/02/19	09:24	Santo Antão	287

Hyperiid amphipods *Phronima* Latreille, 1802 and *Hyperia* Latreille, 1823 were the most common associates with living *P. atlanticum* (Fig. 4F). Smaller hyperiids were frequently seen as white dots on the pyrosomes (Figs. 4D, E; 5A, H; i.e. about the same size as a pyrosome zooid ~ 3 mm, Fig. 5B,C), which on closer inspection had excavated individual zooids to take their place in the colony. These hyperiids were juveniles of an unknown oxycephalid (Fig. 5F,H), most closely related to *Streetsia* Stebbing, 1888 and *Leptocotis* Streets, 1877*.* More specific identification was not possible due to absence of similar sequences and the early development stage of the specimens, which lack established taxonomic characteristics. In addition, several *Phronima* were observed feeding on, or having fashioned their barrel from *P. atlanticum* (Fig. 5I,J)*.* In one case*,* the *Phronima* was left with the pyrosome it was feeding on overnight in chilled seawater and within eight hours it had consumed nearly 20 mm^2^ of the colony (Fig. 5G). Barcoding revealed that two of the six collected *Phronima* were *Phronima sedentaria* (Forskål 1775) and eight barrels were confirmed as pyrosome tissue.

On the seabed, an unidentified decorator crab (382 m, Fig. 4G), five Anomura (361 m, Fig. 4H), two Actiniaria (287 m, Fig. 4I,J), a gastropod (307 m) and five *Pandalopsis* Bate, 1888 (230 m, Fig. 4L) were seen consuming pyrosome carcasses. Two specimens of *Primno* Guérin-Méneville, 1836 were observed and collected with a *P. atlanticum* colony lying on the sea floor (312 m). This hyperiid generally fits the description of *Primno evansi* Sheader, 1986 but barcodes indicate they belong to an as yet undocumented/unsequenced species of *Primno*. In addition, a *Dondice* Er. Marcus, 1958 nudibranch (315 m, Fig. 4K) was observed moving over a colony, although a trophic interaction could not be confirmed.

## Discussion

### Abundance and organic carbon flux

Our statistical analyses showed that increased pyrosome abundances were correlated with higher chl-*a* concentrations and reduced surface temperatures. This suggests that the island- and eddy-induced upwelling surrounding the Cabo Verde landmasses created a favorable environment for *P. atlanticum* blooms in 2018 and 2019. Even though temperature proved to be a less strong predictor of abundance than chl-*a*, both environmental variables are inversely linked in upwelling events*.* Our observations are further confirmed by the fact that pyrosomes have rarely been caught in the oligotrophic ocean surrounding the islands, but were observed in the equatorial upwelling at 23°W and in a cyclonic eddy east of the archipelago (Hauss, unpublished data). Productivity as a driver for pyrosome proliferation was also confirmed by Henschke et al*.*^10^, who investigated *P. atlanticum* abundance in three eddies in the Tasman Sea. In contrast, Schram et al.^44^ reported increased *P. atlanticum* abundance in temperatures associated with reduced phytoplankton productivity in the northern California Current (NCC, i.e. 12–15 °C). However, the latter authors only measured chl-*a* concentrations at 5 m depth, whereas this study and Henschke et al.^10^ measured chl-*a* in the upper 100 m of the water column. When assessing the impact of pyrosome blooms, both local productivity (e.g. upwelling events) and temperature appear to be important factors in determining their abundance and concomitant effect on ocean ecosystems.

*Pyrosoma atlanticum* colonies were found to vertically migrate from the surface at night to depths of approximately 360 m during the day. The highest pyrosome densities were recorded during the night (1.61 colonies m^−3^) and were within the range of reported maximum concentrations in the Atlantic Ocean near the Guinea Dome (0.1–2 colonies m^−3^)^45^, but much lower than densities recorded off the Congo River mouth (9.5–41 colonies m^−3^)^14^ and Oregon coast in 2017 (i.e. NCC, up to 5 colonies m^−3^)^44^. In contrast, the Cabo Verde blooms greatly exceed the maximum densities reported in the Mediterranean Sea (0.187 colonies m^−3^)^46^, the Oregon coast in 2018 (i.e. NCC, 0.07–0.7 colonies m^−3^)^38^ and a cyclonic eddy in the Tasman Sea (0.003 colonies m^−3^)^10^.

The pyrosome abundance measurements in our study differed slightly with sampling gear. PELAGIOS probably provided a slightly higher resolution, considering the continuous transects and wide field of view of the PELAGIOS camera versus the small multinet opening^36^. The tools nicely complemented each other as the multinet allowed us to collect specimens while PELAGIOS showed more detailed depth distributions and biological associations that would have otherwise been missed with the multinet. Our study thus illustrates the need for congruent collection of in situ observations and net samples.

On average, *P. atlanticum* in the Cabo Verde region traveled greater vertical distances (~ 313 m) when compared to *P. atlanticum* in the North Pacific (~ 60 m)^11^ and eddies in the Tasman Sea (~ 222 m)^10^. The diel migration near the Cabo Verde Islands, however, was shallower than the ranges recorded in the Ligurian Sea (410–515 m)^46,47^ and off the Canary Islands (650 m)^48^. It is possible that such regional variation is caused by differences in sunlight attenuation^49^, although food availability, predation, temperature and oxygen concentrations are also known to affect migration ranges^49,50^. We here report one of the deepest records of pyrosomes in the pelagic realm yet, with *P. atlanticum* observed between 1300 and 2500 m depth. Even though these pyrosomes appeared to be alive due to their healthy coloration, they were abnormally shaped when compared to their mesopelagic counterparts. We cannot conclude whether these malformations resulted in some sort of swimming dysfunction that resulted in their abnormal depth, or if the colonies were simply moribund and sinking to the deep.

It is particularly interesting that the highest *P. atlanticum* daytime abundance corresponded to the depth with the lowest oxygen concentrations in the OMZ. Various species of gelatinous zooplankton have been reported to tolerate such low oxygen environments^51–54^, even though these low concentrations make the OMZ inaccessible for many animals^55^. Purcell et al.^51^ suggested that some species of gelatinous zooplankton use the OMZ to relieve predation pressure^18^. At the same time parasitism may also be reduced by temporary visits in low oxygen regions. Nevertheless, pyrosome migration depth may be independent from oxygen, as *P. atlanticum* in the North Pacific was not found to migrate as deep as the OMZ centered around 700 m depth.

During the night, we estimated that *P. atlanticum* released a substantial amount of fecal carbon within the mixed layer (14.75 to 485.57 mg C m^−2^ day^−1^). These rates are somewhat lower than, but within the range of, values estimated in the Atlantic Ocean near the Congo River mouth (87.4 to 1035 mg C m^−2^ day^−1^)^14^. Drits et al.^14^ hypothesized that most of this carbon would be remineralized in the upper water column, as they showed that *P. atlanticum* fecal pellets degrade relatively quickly in warm surface waters (60% of their carbon content was lost after incubation for 45 h at 23 °C). We calculated that at least 13% of the fecal pellets produced (1.96–64.55 mg C m^−2^ day^−1^) were actively transported below the mixed layer by migrating colonies. Considering the relatively fast sinking rate of pyrosome fecal pellets (70 m⋅day^‑1^)^14^ and the immediate temperature drop below the mixed layer to approx. 14–19 °C (slowing microbial degradation), this suggests a substantial flux of organic carbon during pyrosome blooms. Although we only provide an estimate of the fecal export flux, our values seem highly probable as they are within the range of the fecal pellet flux reported for salps in the Sargasso Sea (8.5–137 mg C m^−2^ day^−1^)^56^.

We estimated that the *P. atlanticum* population released an additional amount of 0.0003 to 0.0393 mg C m^−2^ day^−1^ in the form of respiratory carbon once pyrosomes had reached their daytime depth. Even though this flux exceeded the respiratory flux for *P. atlanticum* reported in the Tasman Sea by several orders of magnitude (0.00006–0.12 10^–3^ mg C m^−2^ day^−1^)^10^, it only contributes a fraction to the respiratory flux estimated for zooplankton communities in the North Atlantic near the Canary Islands (3.4 mg C m^−2^ day^−1^)^57^, and micronekton and zooplankton southeast of the Cabo Verde Islands (22.2 mg C m^−2^ day^−1^)^58^. In spite of this, the combined fecal and respiratory flux are over three times higher than the particulate organic carbon flux reported in the ocean surrounding the Cabo Verde Islands (averaging between 9.3–18.1 mg C m^−2^ day^−1^ at 200 m)^59^. The excretory flux, as a result of *P. atlanticum* blooms, thus accounts for a considerable pulse of organic carbon released to deeper water layers.

The detection of pyrosome eDNA down to 1000 m depth confirmed the downward transport of pyrosome material well below their normal depth distribution. At present, we cannot determine if this material originated from fecal material or sinking colonies, but it does show that large pyrosome blooms may affect water layers deeper than their relatively shallow migration range. Since the analysis of eDNA only provides qualitative results, comparison to the quantitative net and video observations is limited to the presence or absence of pyrosome material at certain depths. Future research could be focused on quantifying the amount of pyrosome eDNA through quantitative PCR in relation to bloom density to further substantiate the extent of the downward transport. Nevertheless more specific primers are required, as the custom primers used for the *Phronima* barrels were not specific enough to amplify the pyrosome genetic material in the eDNA samples. In addition, the primers used for eDNA analysis only amplified a short region and were not as specific as desired. Despite Blastn matches to *P. spinosum*, *P. verticillata* and *P. godeauxi,* we assume that all eDNA detected in this study belonged to *P. atlanticum* because of its high abundance in the sampled area and since the former species have not been reported in the eastern tropical North Atlantic^9,13^.

In addition to the organic carbon excreted by living *P. atlanticum*, colony mortality contributed actively to local bentho-pelagic coupling. The carbon deposited by these pyrosome-food falls (0.64 to 41.71 mg C m^−2^) exceeded depositions of pyrosomes along the continental margin of the Mediterranean (0.3 to 1.4 mg C m^−2^)^16^, but were substantially lower than those observed on the continental slope off the Ivory coast, which were reported to average > 5000 mg C m^−2^ (with maximum values reaching 22,000 mg C m^−2^)^15^. In the present and latter studies, pyrosome carcasses generally accumulated along the continental and islands slopes, especially in regions with increased geographic complexity, which acted as a ‘trap’ for sinking colonies^15,16^. Considering that most pyrosomes on the benthos appeared healthy in coloration, with many seen floating a few meters above the seabed, it is possible that colonies were not actually moribund, but were blocked by the island slope during their diel vertical migration. This obstruction of diel vertical migrators by bathymetric features is a well-known phenomenon of seamounts and has been termed “topographic blocking”^60,61^.

### Biological associations

While pyrosomes are known to be prey for a range of oceanic predators, few studies have investigated the biological interactions between pyrosomes and other members of the pelagic food web via in situ observations^23^. Here, we estimated that *P. atlanticum* formed an abundant substrate in the water column (reaching up to 2820 cm^2^ substrate area per m^2^). In particular, *Pyrosoma atlanticum* formed an important substrate for hyperiid amphipods, which are common associates of gelatinous zooplankton (e.g.^62,63^). *Hyperia* amphipods are most often associated with various medusae and ctenophores^64^, with this being the first report of an association with pyrosomes. The juvenile oxycephalids appeared to have a species-specific association with their pyrosome host as they all belonged to a single species and were encountered on pyrosomes from different locations. Moreover, the oxycephalids did not leave the host even with strong physical stimuli. Though most often associated with ctenophores^64,65^, oxycephalids have been reported from a diversity of other gelatinous zooplankton including cnidarians^66^, salps and heteropods^63^, yet this is the first report of any oxycephalid associated with pyrosomes.

*Phronima* are typically considered parasites of pelagic tunicates and are well known for the “barrels” they create from the bodies of their hosts to house their developing young^67^. *Phronima* is primarily found on salps, with few reports of phronimids living or feeding on siphonophores, medusae, and ctenophores^66,68,69^. *Phronima* leave minimal clues to the origin of their barrels, as they remove zooids and smooth the surface. Most barrels in this study could only be identified with the help of DNA barcoding and were found to be constructed from pyrosome tissue.

The fishes we observed inside a pyrosome colony may belong to the genus *Tetragonurus* Risso, 1810 (squaretails), which are known to occur in the Atlantic and reported to occupy the body cavities of salps and pyrosomes^70^. In laboratory experiments, squaretails preferred pelagic tunicates over a variety of different gelatinous zooplankton, a result substantiated by field observations and stomach content analysis^70^. In the case of pyrosomes, Janssen and Harbison^70^ observed squaretails to enter colonies through the shared aperture and to easily exit by swimming backward or forward. In addition to using the colony as shelter, squaretails micropredate on pyrosomes by biting off small pieces and also consume hyperiid amphipods. This would make the interaction partly mutualistic, as the fish can free the colony of its hyperiid parasites. A medusafish, *Icichthys lockingtoni* Jordan and Gilbert, 1880, was also reported inside a *P. atlanticum* colony^21^, although this species has not been reported in the North Atlantic.

To our knowledge this is the first published report of the medusa *Drymonema gorgo* in the Cabo Verde region, and of this species or any *Drymonema* scyphozoan feeding on *P. atlanticum*. Medusozoa are known predators of pelagic tunicates but the available observations mostly involve predation on salps^18,71^. It is possible that *D. gorgo* passively caught the pyrosomes during their upward diel migration, which was also suggested as a hunting strategy for several siphonophores and ctenophores^72,73^.

The benthic invertebrates feeding on *P. atlanticum* in this study were similar to the taxa observed by Lebrato et al*.*^15^ off the Ivory Coast, with exception of the *Dondice* nudibranch that was moving across the colony. Even though we did not see these mollusks feeding on pyrosomes, other nudibranchs are commonly reported to prey on other species of gelatinous zooplankton^74^ and a similar association may apply here.

## Conclusion

The present results illustrate the important ecological role of *P. atlanticum* in the ocean surrounding the Cabo Verde archipelago, impacting both pelagic and benthic ecosystems. Local island-induced upwelling appeared to favor pyrosome proliferation, and it was estimated that a substantial amount of organic carbon was transported to depth via *P. atlanticum*’s fecal pellet production and carcass deposition. Moreover, pyrosomes are here shown to be important components of the Cabo Verde pelagic community during their bloom formation, functioning as a food source and biological substrate in the midwater column. Our study emphasizes the need to complement quantitative net catches with in situ observations to increase spatial resolution, and to allow biomass estimates with additional information on behavior and interactions.

## Supplementary Information

Supplementary Information.

## Data Availability

All data is available on the PANGAEA ® Data Publisher database https://doi.pangaea.de/10.1594/PANGAEA.918915.
