# Supplementary material for: Distribution, associations and role in the biological carbon pump of Pyrosoma atlanticum (Tunicata, Thaliacea) off Cabo Verde, NE Atlantic
Source: Sci Rep. 2021 Apr 29;11:9231. doi: 10.1038/s41598-021-88208-5 (PMC8084940; doi:10.1038/s41598-021-88208-5)
Supplement: Supplementary file 1 — Supplementary Information. [file 41598_2021_88208_MOESM1_ESM.pdf]

Distribution, associations and role in the biological carbon pump of *Pyrosoma atlanticum* (Tunicata, Thaliacea) off Cabo Verde, NE Atlantic

Vanessa I. Stenvers[1,2,3*], Helena Hauss[2], Karen J. Osborn[3,4], Philipp Neitzel[2], Véronique Merten[2], Stella Scheer[2], Bruce H. Robison[4], Rui Freitas[5], Henk-Jan T. Hoving[2*]

[1] Faculty of Science and Engineering, University of Groningen, Nijenborgh 4, 9747 AG Groningen, The Netherlands

[2] GEOMAR, Helmholtz Centre for Ocean Research Kiel, Düsternbrooker Weg 20, 24105 Kiel, Germany

[3] Department of Invertebrate Zoology, National Museum of Natural History, Smithsonian Institution, Washington, DC 20013, U.S.A.

[4] Monterey Bay Aquarium Research Institute, 7700 Sandholdt Road, Moss Landing, CA 95039-9644, U.S.A.

[5] Institute of Engineering and Marine Sciences, Atlantic Technical University, CP 163, Mindelo, Cabo Verde

*Corresponding authors: Vanessa I. Stenvers (v.i.stenvers@gmail.com), Henk-Jan T. Hoving (hhoving@geomar.de)

Keywords: carbon transport, environmental DNA, Diel Vertical Migration, oxygen minimum zone, deep sea

SUPPLEMENTARY METHODS

PYROSOMA ATLANTICUM LENGTH AND WET WEIGHT

In 2018, pyrosome length and weight were quantified on board, after which samples were stored in ethanol or formalin for later reference. In 2019, samples from the Fogo station were weighed in the laboratory ashore after preservation in formalin, while the samples collected in the eddy were measured onboard the research vessel. To compare all samples, the latter size and weight measurements needed to be converted to the original wet weight (WW) values, since chemical preservation is known to reduce tissue weight[1]. Here, we applied a 65.8% loss of WW reported by Pakhomov[1] for *Pyrosoma* sp. preservation, and a formula by Henschke *et al.*[2] to estimate the WW (g) from the pyrosome total length (TL in mm):

$$WW = 0.0013TL^2 + 0.0151TL \quad (S1)$$

To estimate the colony width from pyrosome lengths, the length to width ratio was calculated from photographed multinet specimens with ImageJ v1.52k[3]. For a conservative estimate, width in these specimens was measured near the closed end of the colony (below the curved the apex) where the width was most narrow. The length to width ratio was 1 to 0.25 ± 0.04 (s.d., n=16). The substrate area of *P. atlanticum* was calculated from the average colony length and width per station, according to the lateral surface area of a cylinder. These values were then multiplied by the depth-integrated multinet abundance to obtain the substrate area (cm^2) per surface water area (m^2).

GENETIC SEQUENCING

Genetic analysis of the crustaceans found on pyrosomes was performed in the Smithsonian National Museum of Natural History Laboratory of Analytical Biology (LAB). Pyrosome eDNA and *Phronima* barrels were analyzed at the GEOMAR Helmholtz Centre for Ocean Research Kiel.

For the crustaceans (n=35), total genomic DNA was extracted from tissue samples using the AutoGenPrep 965 high-throughput DNA extractor (AutoGen) following the manufacturer's protocols for animal tissue extraction. This included two ethanol wash steps and a final elution in 100 µl of AutoGen R9 reagent solution. A ~655 bp region at the 5' end of cytochrome oxidase-*c* subunit I (COI) was amplified using primers jgLCO1490 and jgHCO2198[4]. Polymerase chain reaction (PCR) was carried out in 10 µl reactions for each sample, consisting of 5 µl GoTaq® Mastermix (2X; Promega Inc., Madison, WI), 3.2 µl sterile water, 0.3 µl of each forward and reverse primers (10mM concentration), 0.1 µl Magnesium

Chloride (50mM concentration) , 0.1 µl BSA (New England Biolabs, 20 mg/ml) and 1 µl template DNA. The PCR thermocycling protocol consisted of the following steps: initial denaturation at 95°C for 5 min; 4 cycles of 94ºC for 30 s, 50°C for 45 s and 72°C for 60 s; then 34 cycles of 94ºC for 30 s, 45°C for 45 s and 72°C for 60 s; and the final extension at 72°C for 8 min. PCR products were visualized using 1.5% agarose gel electrophoresis and purified with USB ExoSAP-IT following the manufacturer's protocol (Affymetrix, Santa Clara, CA). Purified PCR products were then used in cycle sequencing with BigDye® Terminator (Life Technologies, Carlsbad, CA) using the following thermal cycling profile: 4 min at 96 °C, 30 cycles of 10 s at 95 °C, 30 s at 50 °C, and 4 min at 60°C. Purification of cycle sequencing products was performed with Sephadex® G-50 gel column filtration (GE Healthcare Life Sciences, Pittsburgh, PA). Purified PCR products were sequenced using a 3730xl DNA analyzer (Applied Biosystems, Inc., Waltham, MA).

For the pyrosome eDNA (n=24), DNA was extracted with help of the Qiagen DNeasy Blood and Tissue Kit (Qiagen Inc., Valencia, CA, USA) with a modified protocol. 720 µl ATL-Buffer and 80 µl proteinase K were inserted directly into the filter and incubated at 56°C for at least 2 hours with agitation. The buffer mix was transferred to new tubes and 600 µl of each sample mixed with 600 µl AL-Buffer and 600 µl 99% Ethanol. After this step, the standard DNeasy Blood and Tissue instructions were followed. The same kit was used for the extraction of DNA from *Phronima* barrel tissue (n=8) according to manufacturer's instructions. Two regions of the 18S rRNA gene were amplified using the custom forward 5'-CCG TGC TCA ATG GTG ACT CT-3' and reverse 5'-TAA GAA CCT CGG TAG GCG GA-3' primers for the eDNA (~30 bp), and the forward 5'-GGC CGT TCT TAG TTG GTG GA-3' and reverse 5'-TTG CTC AAT CTC GTG TGG CT-3' primers for the *Phronima* barrels (~150 –200 bp). PCR reactions were carried out with the TaqMan Environmental Master Mix 2.0, following the manufacturer's instruction for a 25 µl reaction volume, including 10 µl master mix, 8 µl sterile water, 1 µl of each forward and reverse primers and 5 µl DNA template. For the pyrosome eDNA, the thermocycling included 95°C for 10 min; 35 cycles of 94ºC for 30 s, 66°C for 30 s and 72°C for 60 s; and the final extension at 72°C for 5 min. For the *Phronima* barrels, the thermocycling protocol was as follows: 95°C for 10 min; 32 cycles of 94ºC for 30 s, 67°C for 30 s and 72°C for 60 s; and the final extension at 72°C for 5 min. All PCR products were visualized using 1.2% agarose gel electrophoresis. Samples were sent to the Institute of Clinical Molecular Biology (IKMB, Germany) for purification and sequencing on an Applied Biosystems 3730xl DNA Analyzer.

For the crustacean and *Phronima* barrel sequences, forward and reverse raw traces were assembled into contigs using the 'De Novo Assemble' function in Geneious v9 or v11 and Geneious Prime 2020.1.1 (Biomatters Ltd., Auckland, New Zealand), where primers and poor quality 3' and 5' ends were automatically trimmed. The eDNA sequences were assembles using CodonCode Aligner v. 3.7.1.2. All sequences produced in the current study were uploaded to GenBank as part of NCBI BioProject xxx (https://www.ncbi.nlm.nih.gov/bioproject/XXX).

REFERENCES

1 Pakhomov, E. Correction of zooplankton and benthos biomass underestimations from formaldehyde-preserved samples. *Archive of Fishery and Marine Research* **50**, 141-148 (2003).

2 Henschke, N. *et al.* Large Vertical Migrations of *Pyrosoma atlanticum* Play an Important Role in Active Carbon Transport. *Journal of Geophysical Research: Biogeosciences* **0**, doi:10.1029/2018jg004918 (2019).

3 Schneider, C. A., Rasband, W. S. & Eliceiri, K. W. NIH Image to ImageJ: 25 years of image analysis. *Nature Methods* **9**, 671-675, doi:10.1038/nmeth.2089 (2012).

4 Geller, J., Meyer, C., Parker, M. & Hawk, H. Redesign of PCR primers for mitochondrial cytochrome c oxidase subunit I for marine invertebrates and application in all-taxa biotic surveys. *Molecular Ecology Resources* **13**, 851-861, doi:10.1111/1755-0998.12138 (2013).

SUPPLEMENTARY FIGURES

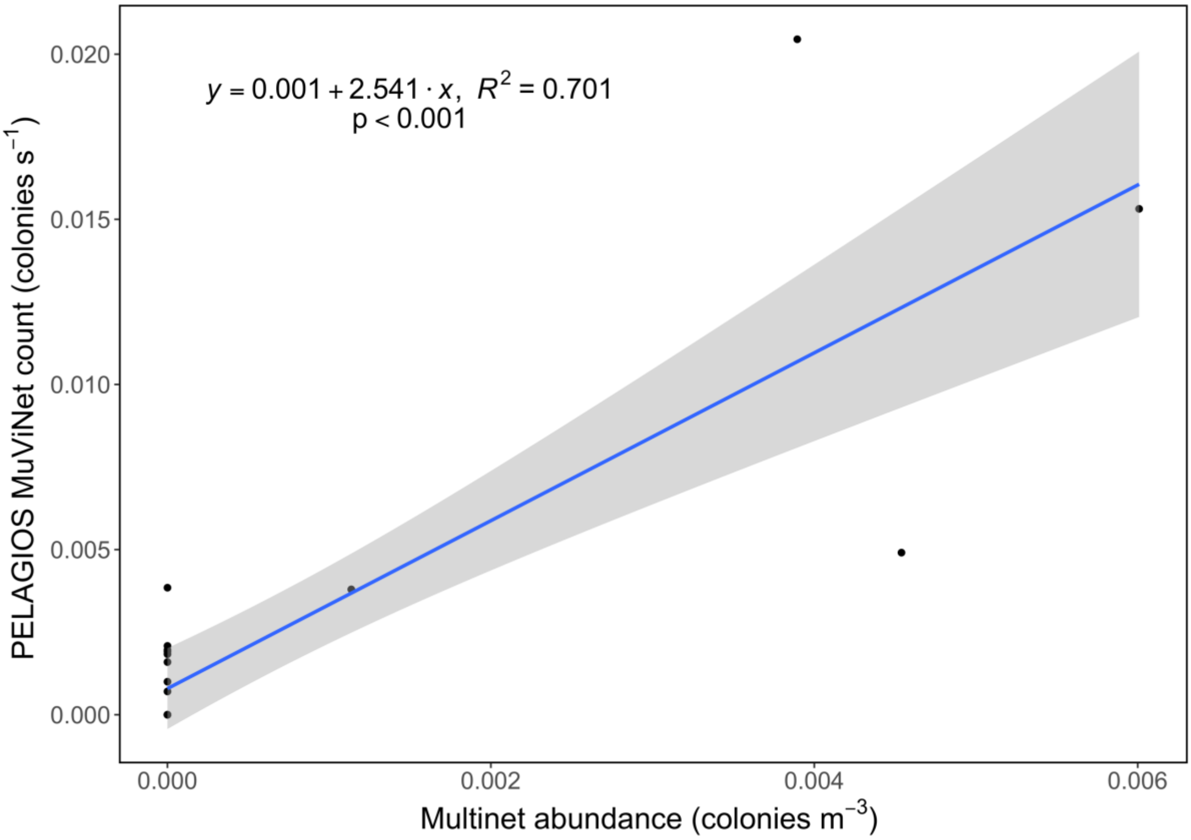

Supplementary Figure S1. Counts of *P. atlanticum* with the PELAGIOS camera mounted on the multinet (i.e. MuViNet) as a function of the corresponding multinet catches. MuViNet observations/catches were made at the Fogo and Eddy station in 2018. The blue line represents the linear model fitted to the data with a 95% confidence interval in grey. The standard error for the intercept is 0.0006 and for the slope 0.35.

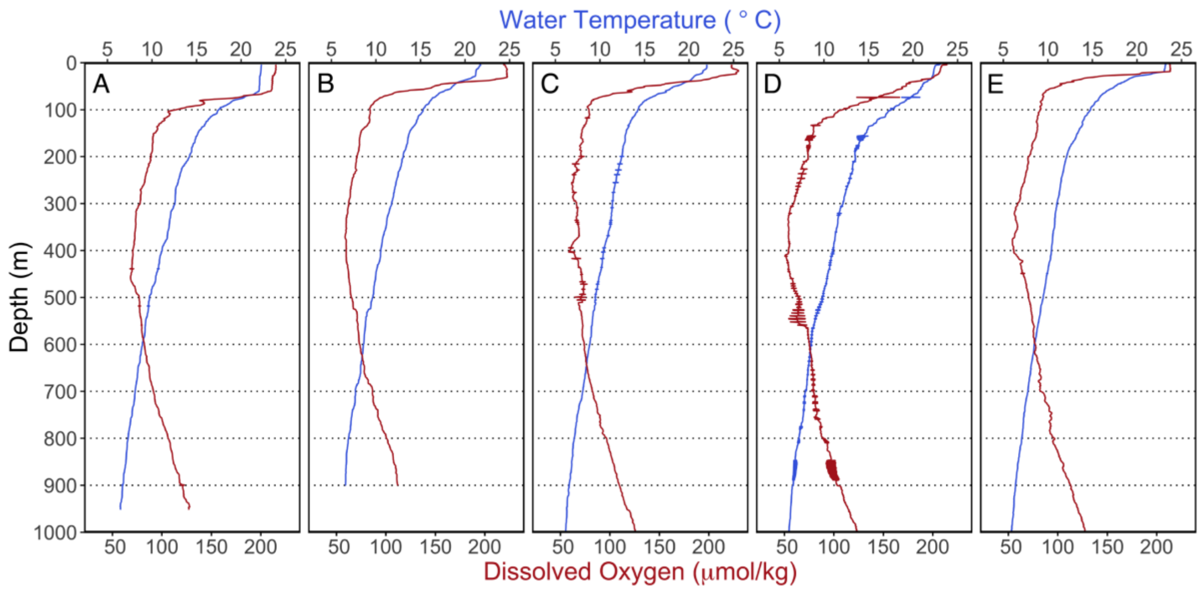

Supplementary Figure S2. Temperature (°C; blue) and dissolved oxygen (µmol/kg; red) profiles in the eastern tropical North Atlantic, in 2018 (**A-C**) and 2019 (**D-E**). Measurements in 2018 were taken at (**A**) Santo Antão, (**B**) Fogo and (**C**) a cyclonic eddy, and in 2019 at (**D**) Fogo and (**C**) a second cyclonic eddy.

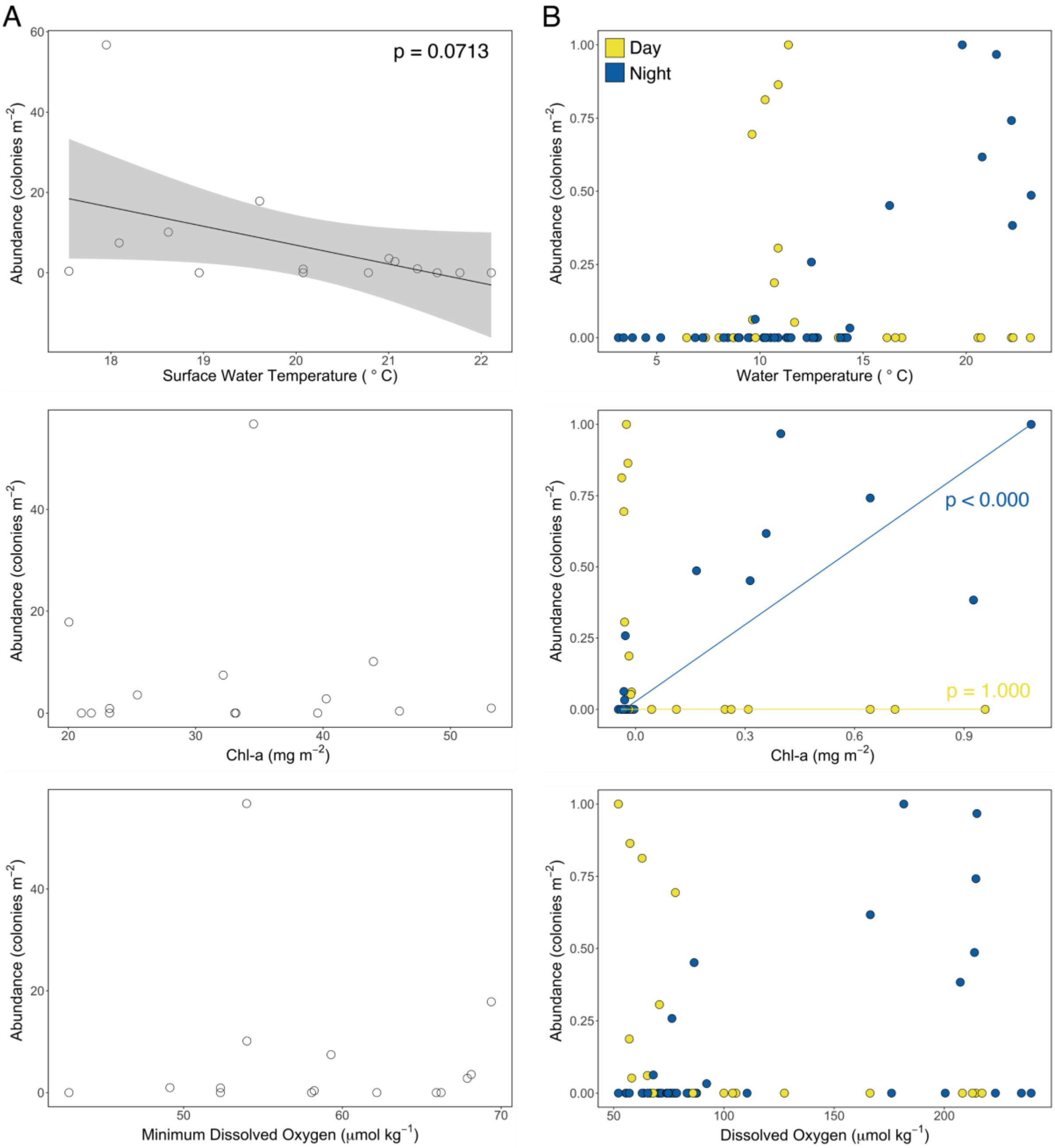

Supplementary Figure S3. Scatter plot of *Pyrosoma atlanticum* integrated abundance in relation to water temperature, chlorophyll-*a* and dissolved oxygen concentrations. (**A**) Data points per station, showing linear regression fit and p-value for most parsimonious model containing surface water temperature (adjusted R^2= 0.170). (**B**) Data points per depth bins of the multinet, showing quantile regression fit of most parsimonious model with day (yellow) and night (blue) observations as fixed factor and corresponding p-values.

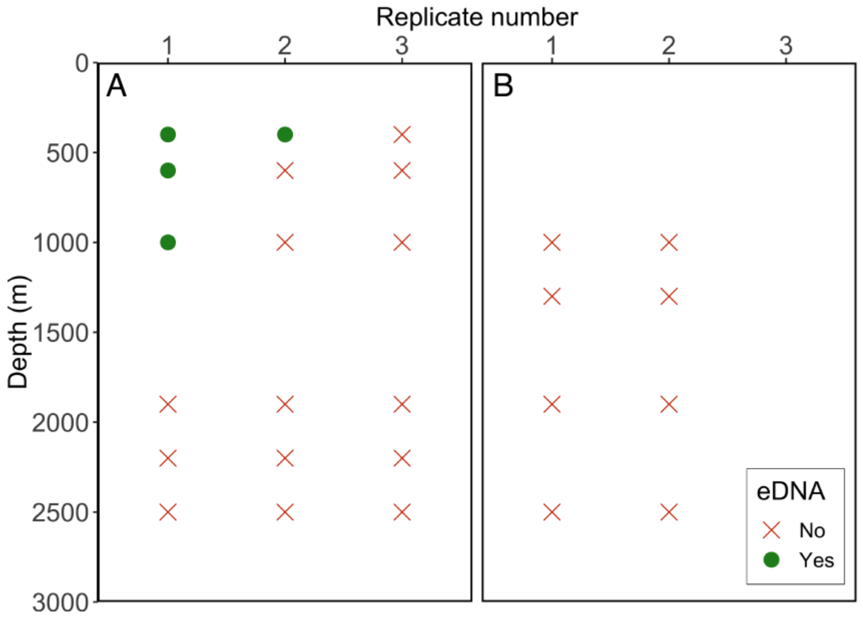

Supplementary Figure S4. Detection of *P. atlanticum* environmental DNA (eDNA) at various depths in the (**A**) cyclonic eddy and (**B**) at the Cabo Verde Ocean Observatory (CVOO) in 2019. Green circles indicate presence of pyrosome eDNA, red crosses indicate absence.

SUPPLEMENTARY TABLES

Supplementary Table S1. Date, time and depth intervals of multinet deployment for day- and nighttime sampling. SA and SAO are short for the Santo Antão coastal and oceanic stations, respectively.

Station	Date	Time	Depth intervals (m)
SA	17/02/18	23:45	1030 – 900 – 700 – 550 – 450 – 350 – 250 – 150 – 50 – 0
	18/02/18	13:38	1030 – 900 – 700 – 550 – 450 – 350 – 250 – 150 – 50 – 0
SAO	20/02/18	18:25	2500 – 2350 – 2050 – 1750 – 1450 – 1150 – 600 – 300 – 100 – 0
Fogo	23/02/18	12:00	950 – 900 – 700 – 550 – 450 – 350 – 250 – 150 – 75 – 0
	23/02/18	23:00	950 – 900 – 700 – 550 – 450 – 350 – 250 – 150 – 75 – 0
Eddy	25/02/18	21:25	1000 – 900 – 700 – 550 – 450 – 350 – 250 – 150 – 75 – 0
CVOO	28/02/18	04:00	950 – 900 – 700 – 550 – 450 – 350 – 250 – 150 – 75 – 0
	28/02/18	10:33	950 – 900 – 700 – 550 – 450 – 350 – 250 – 150 – 75 – 0
SA	11/02/19	14:11	900 – 800 – 700 – 600 – 500 – 400 – 300 – 200 – 100 – 0
	11/02/19	19:31	900 – 800 – 700 – 600 – 500 – 400 – 300 – 200 – 100 – 0
Fogo	15/02/19	10:57	900 – 700 – 600 – 500 – 400 – 300 – 200 – 100 – 50 – 0
	15/02/19	20:18	900 – 700 – 600 – 500 – 400 – 300 – 200 – 100 – 50 – 0
Fogo oceanic	16/02/19	12:03	900 – 700 – 600 – 500 – 400 – 300 – 200 – 150 – 50 – 0
Eddy	22/02/19	13:58	600 – 500 – 450 – 400 – 300 – 200 – 150 – 100 – 40 – 0
	22/02/19	20:26	600 – 500 – 450 – 400 – 300 – 200 – 150 – 100 – 40 – 0

Supplementary Table S2. Date, time and depth intervals of PELAGIOS deployment for day- and nighttime sampling. SA and SAO are short for the Santo Antão coastal and oceanic stations, respectively. Asterisks and grey depth intervals indicate failed video recordings due to technical failures.

Station	Date	Time	Depth intervals (m)
SA	16/02/18	14:05	50 – 100 – 200 – 300 – 400 – 500 – 600 – 800 – 900
	17/02/18	10:34	50 – 100 – 200 – 300 – 400 – 430 – 500 – 600 – 800 – 900 – 1000
	17/02/18	20:33	50 – 100 – 200 – 300 – 400 – 500 – 600 – 800 – 1000
	19/02/18	13:28	20 – 50 – 100 – 200 – 300 – 380 – 500 – 600 – 800
SAO	20/02/18	10:51	*50 – 200 – 400 – 1000 – 1300 – 1600 – 1900 – 2200 – 2500*
	20/02/18	23:06	*50 – 100 – 200 – 400* – 1000 – 1300 – 1600 – 1900 – 2200 – 2500
Eddy	21/02/18	19:29	50 – 100 – 130 – 140 – 170 – 200 – 250 – 300
Fogo	22/02/18	14:01	50 – 100 – 200 – 300 – 400 – 500 – 600 – 800
	23/02/18	16:25	20 – 50 – 100 – 200 – 300 – 400 – 500 – 600 – 800 – 950
SA	06/02/19	20:34	*30 – 50 – 200 – 300 – 400 – 500 – 600 – 700*
CVOO	13/02/19	13:02	*200 – 300 – 400 – 500 – 600 – 700 – 800 – 900 – 1000 – 1300 – 1600*
Fogo	15/02/19	21:21	30 – 50 – 100 – 200 – 300 – 400 – 500 – 600 – 800 – 875 – 900
Fogo	16/02/19	13:46	200 – 300 – 400– 500 – 600 – 700
Eddy	22/02/19	15:05	30 – 50 – 100 – 200 – 300 – 400 – 500 – 600 – 700
Eddy	22/02/19	21:53	30 – 40 – 50 – 100 – 150 – 200 – 300 – 350 – 400 – 475

Supplementary Table S3. Oceanographic conditions per station, including average sea surface temperature (in the upper 100 m, approximate mixed layer), minimum dissolved oxygen concentration and integrated chl-*a* concentrations (in the mixed layer). SA and SAO are short for the Santo Antão coastal and oceanic stations, respectively.

Station	Year	Surface temperature (Day – Night °C)	Min. dissolved oxygen (Day – Night µmol·kg^{-1})	Chl-a (Day – Night mg·m^{-2})
SA	2018	21.0 – 21.1	67.9 – 68.1	25.4 – 40.2
SAO	2018	19.6 – 21.4	69.3 – 69.4	20.1 – 35.6
CVOO	2018	20.8 – 21.5	65.9 – 66.2	21.0 – 33.1
Fogo coastal	2018	18.1 – 19.0	58.1 – 59.3	32.2 – 33.2
Eddy	2018	17.5 – 18.9	57.2 – 58.2	39.1 – 46.0
SA	2019	21.8 – 22.1	42.8 – 62.2	21.8 – 39.6
CVOO	2019	21.2 – 21.6	52.5 – 54.0	16.4 – 25.8
Fogo coastal	2019	20.1 – 21.3	49.1 – 52.3	23.2 – 53.2
Fogo oceanic	2019	20.1 – 21.3	46.6 – 52.3	23.2 – 25.7
Eddy	2019	18.0 – 18.6	54.0 – 54.0	34.5 – 44.0

Supplementary Table S4. Environmental drivers of *Pyrosoma atlanticum* spatial and vertical depth distribution according to linear and median quantile regression.

Coefficients	Estimate/Value	Standard error	t-value	p-value
**Linear regression**				
Intercept	101.040	48.154	2.098	0.056
Water temperature	-4.708	2.398	-1.964	0.071
**Median quantile regression**				
Intercept	0.000	0.002	0.000	1.000
Chl-*a*	0.000	0.006	0.000	1.000
Day vs Night	0.028	0.003	8.970	< 0.000
Chl-*a* vs Night	0.897	0.013	72.048	< 0.000

Supplementary Table S5. Multinet catches of *P. atlanticum* with weighed mean depth (WMD), average length, biomass, estimated fecal pellet production (FP) per station during the night, the amount of FP transported below the mixed layer (FP ML) and the respiratory carbon released below the mixed layer (Respiratory C ML). SA and SAO are short for the Santo Antão coastal and oceanic stations, respectively.

Station	Date	Time (hrs)	WMD (m)	Average length (cm)	Biomass (g WW m^{-2})	FP (mg C m^{-2} n^{-1})	FP ML (mg C m^{-2} d^{-1})	Respiratory C ML (mg C m^{-2} d^{-1})
SA	17/02/18	23:45	32.70	5.90 ± 2.80	28.75	99.79	13.27	
SA	18/02/18	13:38	473.42	6.66 ± 2.55	53.72	–	–	0.0041
SAO	20/02/18	18:25	68.01	5.12 ± 1.29	85.14	295.51	39.29	0.0053
CVOO	28/02/18	04:00	–	–	0	–	–	
CVOO	28/02/18	10:33	–	–	0	–	–	
Fogo coastal	23/02/18	12:00	402.29	5.29 ± 1.46	49.17	–	–	0.0031
Fogo coastal	23/02/18	23:00	–	–	0	–	–	
Eddy	25/02/18	21:25	32.50	7.44 ± 6.01	4.25	14.75	1.96	0.0003
SA	11/02/19	14:11	–	–	0	–	–	
SA	11/02/19	19:31	–	–	0	–	–	
Fogo coastal	15/02/19	10:57	200.00	6.80 ± 1.66	6.59	–	–	0.0004
Fogo coastal	15/02/19	21:18	48.73	6.06 ± 0.24	5.51	19.12	2.54	
Fogo oceanic	16/02/19	12:03	–	–	0	–	–	
Eddy	22/02/19	13:58	360.54	7.95 ± 1.45	542.55	–	–	0.0393
Eddy	22/02/19	20:26	46.98	9.22 ± 2.11	139.90	485.57	64.55	

Supplementary Table S6. Depth-integrated abundance and substrate area of *P. atlanticum* aggregations, quantified with multinet and/or PELAGIOS. SA and SAO are short for the Santo Antão coastal and oceanic stations, respectively. Asterisks indicate failed video recordings due to technical complications, hyphens indicate no deployment of gear.

Station	Year	Multinet (col m^{-2})		Pelagios (col m^{-2})		Multinet (col surface cm^2 m^{-2})	
		Day	Night	Day	Night	Day	Night
SA	2018	3.57	2.81	40.66	11.90	124.5	76.9
SAO	2018	-	17.85	*	*	-	368.0
CVOO	2018	0.0	0.0	-	-	0.0	0.0
Fogo coastal	2018	7.45	0.0	110.51	8.37	163.9	0.0
Eddy	2018	-	0.39	-	13.94	-	17.0
SA	2019	0.0	0.0	-	*	0.0	0.0
CVOO	2019	-	-	*	-	-	-
Fogo coastal	2019	0.91	0.98	13.54	1.77	33.1	28.3
Fogo oceanic	2019	0.0	-	-	-	0.0	-
Eddy	2019	56.75	10.12	1.07	15.93	2820.4	676.5

Supplementary Table S7. Top blastn matches to pyrosome eDNA in the cyclonic eddy

Depth	Name	E-value	Identity	Accession number
400 m	Pyrosoma atlanticum	9.00E-32	100.00%	HQ015385.1
	Pyrosoma godeauxi	9.00E-32	100.00%	HQ015384.1
	Pyrosomella verticillata	9.00E-32	100.00%	HQ015383.1
	Pyrosomella verticillata	9.00E-32	100.00%	HQ015382.1
	Pyrosoma atlanticum	9.00E-32	100.00%	HQ015381.1
	Pyrosomella verticillata	9.00E-32	100.00%	HQ015380.1
	Pyrostremma spinosum	9.00E-32	100.00%	HQ015379.1
	Pyrosomella verticillata	9.00E-32	100.00%	FM244863.1
	Pyrosoma godeauxi	9.00E-32	100.00%	FM244862.1
400 m	Pyrosoma atlanticum	9.00E-32	100.00%	HQ015385.1
	Pyrosoma godeauxi	9.00E-32	100.00%	HQ015384.1
	Pyrosomella verticillata	9.00E-32	100.00%	HQ015383.1
	Pyrosomella verticillata	9.00E-32	100.00%	HQ015382.1
	Pyrosoma atlanticum	9.00E-32	100.00%	HQ015381.1
	Pyrosomella verticillata	9.00E-32	100.00%	HQ015380.1
	Pyrostremma spinosum	9.00E-32	100.00%	HQ015379.1
	Pyrosomella verticillata	9.00E-32	100.00%	FM244863.1
	Pyrosoma godeauxi	9.00E-32	100.00%	FM244862.1
600 m	Pyrosoma atlanticum	9.00E-32	100.00%	HQ015385.1
	Pyrosoma godeauxi	9.00E-32	100.00%	HQ015384.1
	Pyrosomella verticillata	9.00E-32	100.00%	HQ015383.1
	Pyrosomella verticillata	9.00E-32	100.00%	HQ015382.1
	Pyrosoma atlanticum	9.00E-32	100.00%	HQ015381.1
	Pyrosomella verticillata	9.00E-32	100.00%	HQ015380.1
	Pyrostremma spinosum	9.00E-32	100.00%	HQ015379.1
	Pyrosomella verticillata	9.00E-32	100.00%	FM244863.1
	Pyrosoma godeauxi	9.00E-32	100.00%	FM244862.1
1000 m	Pyrosoma atlanticum	9.00E-32	100.00%	HQ015385.1
	Pyrosoma godeauxi	9.00E-32	100.00%	HQ015384.1
	Pyrosomella verticillata	9.00E-32	100.00%	HQ015383.1
	Pyrosomella verticillata	9.00E-32	100.00%	HQ015382.1
	Pyrosoma atlanticum	9.00E-32	100.00%	HQ015381.1
	Pyrosomella verticillata	9.00E-32	100.00%	HQ015380.1
	Pyrostremma spinosum	9.00E-32	100.00%	HQ015379.1
	Pyrosomella verticillata	9.00E-32	100.00%	FM244863.1
	Pyrosoma godeauxi	9.00E-32	100.00%	FM244862.1

Supplementary Table S8. Carbon content *P. atlanticum* on seabed, documented by JAGO and OFOS at the Santo Antão (SA) and Fogo stations.

Station	Platform	Date	Transect length (km)	Total nr. of *P. atlanticum*	Carbon content (mg C m^{-2})
SA	JAGO	07/02/2019	0.5	53	3.44
SA	JAGO	10/02/2019	0.3	340	39.75
SA	JAGO	12/02/2019	0.2	4	0.64
SA	OFOS	09/02/2019	0.03	6	10.03
SA	OFOS	11/02/2019	0.05	1	1.17
Fogo	JAGO	17/02/2019	0.3	86	8.87
Fogo	OFOS	18/02/2019	0.08	54	41.71
